# Modelling SARS-CoV-2 infection in a human alveolus microphysiological system

**DOI:** 10.1099/acmi.0.000814.v3

**Published:** 2024-09-11

**Authors:** Tanja Šuligoj, Naomi S. Coombes, Catherine Booth, George M. Savva, Kevin R. Bewley, Simon G.P. Funnell, Nathalie Juge

**Affiliations:** 1 Quadram Institute Bioscience, Norwich Research Park, Norwich, NR4 7UQ, UK; 2 UK Health Security Agency, Manor Farm Road, Porton Down, Salisbury, SP4 0JG, UK

**Keywords:** BSL3, COVID-19, lung-on-chip, microphysiological system, SARS-CoV-2

## Abstract

The coronavirus 2019 pandemic has highlighted the importance of physiologically relevant *in vitro* models to assist preclinical research. Here, we describe the adaptation of a human alveolus microphysiological system (MPS) model consisting of primary human alveolar epithelial and lung microvascular endothelial cells to study infection with SARS-CoV-2 at Biosafety Level 3 facility. This infection model recapitulates breathing-like stretch and culture of epithelial cells at the air–liquid interface and resulted in clinically relevant cytopathic effects including cell rounding of alveolar type 2 cells and disruption of the tight junction protein occludin. Viral replication was confirmed by immunocytochemical nucleocapsid staining in the epithelium and increased shedding of SARS-CoV-2 virus within 2 days post-infection, associated with changes in innate host immune responses. Together, these data demonstrate that, under the experimental conditions used in this work, this human alveolus MPS chip can successfully model SARS-CoV-2 infection of human alveolar lung cells.

## Data Summary

The authors confirm that the data supporting the findings of this study are available within the article and its supplementary materials.

## Introduction

Coronavirus disease (COVID-19) is a severe acute respiratory syndrome (SARS) caused by SARS-CoV-2. It was first reported in December 2019 in Wuhan (China) and is estimated to have caused as many as 24.8 million deaths worldwide so far [1]. In mild or moderate cases, SARS-CoV-2 infects the upper airways, but severe cases tend to be marked by progression of the infection into the lower airways and alveoli [2,3]. In severe COVID-19 cases, disease can develop into progressive respiratory failure, resulting in death due to diffuse alveolar damage, inflammation and pneumonia [4]. The severity of lung injury in COVID-19 pneumonia is associated with elevated levels of circulating cytokines [5].

Approximately 90% of total lung volume is composed of the alveolar region comprising type I (AT1) and type II (AT2) alveolar epithelial cells. AT1 cells are large and flat pneumocytes that are responsible for gaseous exchange, whereas AT2 cells release pulmonary surfactants that cover the alveolar epithelium [6]. AT2 cells have also been reported to be the primary target of SARS-CoV-2 infection [7]. SARS-CoV-2 enters host cells through the binding of the spike protein to the cell receptor, angiotensin-converting enzyme 2 [8]. Neuropilin-1, an integrin binding protein, has also been shown to be an alternative receptor for SARS-CoV-2 host cell entry [9]. SARS-CoV-2 uses the protease activity of the TMPRSS2 protein in the process of entering cells [10].

There are many reported *in vitro* models of human lung available to study SARS-CoV-2 infection. Standard 2D cell cultures have low *in vivo* mimicry, which can be improved by culturing cells on transwells and air–liquid interface [11,12]. However, these models lack microfluidic flow and breathing-like stretch which recapitulate organ-level lung functions [13]. Lung microphysiological system (MPS) is being increasingly recognized as reliable models for studying SARS-CoV-2 infection [3,4, 11, 14]. Mechanically, active lung-on-chips recapitulate multiple physiological functions observed in the breathing lung such as the microarchitecture of the alveolar-capillary unit, the maintenance of alveolar epithelial cells in air–liquid interface (ALI) and physiologically relevant mechanical forces [13]. Physiological breathing motions on lung-on-chips activates protective host innate immune responses to supress viral infection [15], whereas ALI induces cell differentiation and promotes formation of tight junction proteins [12]. SARS-CoV-2 infection of primary human alveolar and primary human lung endothelial cells on chips under microfluidic flow showed that infection resulted in endotheliitis and limited apical release of virions [3]. However, this model lacked the physiologically relevant stretch after infection with the virus. A study using alveolus-on-chips with microfluidic flow and breathing-like stretch motion showed that endothelial cells were more susceptible to infection than epithelial cells. However, this model used pseudovirus, immortalized AT2 cells and umbilical vein endothelial cells [14]. In contrast, in the absence of stretch, SARS-CoV-2 was shown to primarily infect and replicate in the alveolar epithelium using immortalized AT2 cells, while no replication was detected in the endothelium of the model [4].

Here, we used a more physiologically relevant MPS model containing primary human alveolar epithelial cells cultured under ALI conditions and primary human microvascular endothelial cells to study SARS-CoV-2 infection. The model was subjected to microfluidic flow and breathing-like motion with airflow, and the media composition was adapted to more accurately mimic physiological conditions. Using this system, we showed that SARS-CoV-2 successfully infected epithelial cells, resulting in morphological changes typically seen in human cases with subsequent apical release of live SARS-CoV-2 virus and induced human innate host immune response.

## Methods

### Culture of alveolus-on-chips

The establishment of the alveolus-on-chip model is based on Huh *et al*. [5] using Emulate’s Alveolus Lung-Chip Co-Culture Protocol [16] adapted and developed as highlighted below. Briefly, 12 microfluidic two-channel stretchable chips (chip-S1, Emulate Inc, Boston, MA) were activated and coated with extracellular matrix proteins. Primary human lung alveolar epithelial cells (Cell Biologics, H-6053) were seeded on top channel and primary human lung microvascular endothelial cells (Cell Biologics, H-6011) on bottom channel. Chips were connected to the Pods and Zoës (Emulate Inc.) and microfluidic flow in both channels set to 30 µl h^−1^. ALI was set up in the top channel and medium perfused through bottom channel at 30 µl h^−1^. Dexamethasone was omitted from the medium the 1 day after ALI was established. Airflow (30 µl h^−1^) was introduced together with stretch at 2% and 0.2 Hz which was increased to 5% the following day by setting up the required parameters on Zoës (Emulate Inc.) (Fig. S1, available in the online supplementary material).

### Assessment of apparent permeability

Apparent permeability (Papp) assay was performed on alveolus-on-chips four days after ALI was set up following the manufacturer’s instructions specified in the Emulate protocol for barrier function analysis [17]. Briefly, the fluorescent probe fluorescein isothiocyanate (FITC)-dextran with molecular mass of 4 kDa at 100 µg ml^−1^ was added to the basolateral channel and perfused for 4.5 h at a flow rate of 120 µl h^−1^. The apical channel contained medium only. Effluent media from apical and basolateral channels were collected and fluorescence intensity quantified using a FLUOstar Omega plate reader (BMG LABTECH, Ortenberg, Germany).

### SARS-CoV-2 infection and sample preparation for downstream analyses

All experimental work with SARS-CoV-2 was performed in Biosafety Level 3 (BSL3) facility at QIB (Norwich, UK) following the Advisory Committee on Dangerous Pathogens’ guidelines on Management and operation of microbiological containment laboratories. The Australia/VIC01/2020 SARS-CoV-2 isolate, a pre-D614G B-clade virus, was isolated from a man who returned to Australia from Wuhan on 19 January 2020 (EPI_ISL_406844) [18]. This virus has >99.9 % sequence identity with the Wuhan-Hu-1 reference genome (GenBank: MN908947). The virus was a generous donation from the Doherty Institute (Victoria, Australia) and expanded in Vero-hSLAM cells (ECACC, 04091501) as previously described [19].

The virus was diluted in ALI medium without both foetal calf serum (FCS) and dexamethasone, to a multiplicity of infection (MOI) of 1, and the six test chips were exposed apically for 1 h at 37 °C under static conditions (Fig. S1). Six control chips were mock-treated with the medium only. Following 1h incubation, the ALI was re-established in all 12 chips. The basolateral flow of ALI medium (with 2 % FCS and without dexamethasone) was 30 µl h^−1^, apical airflow at 30 µl h^−1^ and stretch at 5 % at 0.2 Hz (Fig. S1).

For cytokine analysis, basolateral effluent media were collected at 6 h post-infection (hpi) and 24 hpi from all chips (six per group), 48 hpi (four chips per group) and 72 hpi (two chips per group) after inoculation with either the virus or mock treatment. Triton X-100 was added at 0.5 % v/v to inactivate the virus. At 24, 48 and 72 hpi two chips from mock and virus treated groups were washed with PBS for subsequent focus forming assay (FFA) and then fixed with paraformaldehyde (PFA) for immunofluorescence analysis.

### Phase contrast microscopy

Representative images of mock-treated and SARS-CoV-2-infected alveolus-on-chips were taken on PFA-fixed chips collected at 24, 48 and 72 hpi. To minimize bias, all images of the chips were acquired in the same area (middle of epithelial channel). Images were acquired using 20× objective and Revolve 4M microscope (Echo, San Diego, USA).

### Immunostaining and microscopy

For AT2 and occludin (OCLN) staining, the epithelial cells were permeabilized with 0.25 % Triton X-100 for 15 min and blocked with 0.3 M glycine, 10 % goat serum and 1 % BSA in PBS with 0.05 % Tween 20. AT2 were stained with anti-HT2-280 antibody (Terrace Biotech, TB-27 AHT2-280) at 1 : 100 and tight junctions with OCLN antibody conjugated with Alexa488 (Thermo Fisher Scientific, 10073504) at 1 : 50 followed by goat anti-mouse IgM secondary antibody conjugated with Alexa594 (Thermo Fischer Scientific, A21044) at 1 : 500. Cell nuclei were counterstained with DAPI (Sigma, D9542). Images were acquired with confocal microscope Zeiss LSM880 (Zeiss, Oberkochen, Germany) using 10× Plan-Apochomat air objective and 20× N-Achroplan water dipping objective. ImageJ software [20] was used for image processing. AT2 and OCLN were imaged in both mock-treated and SARS-CoV-2-infected chips in the middle of the chips at 48 and 72 hpi.

For SARS-CoV-2 nucleocapsid staining, the cells were permeabilized with 1 % saponin in PBS and blocked with 1 % BSA in PBS. SARS-CoV-2 nucleocapsid was stained with rabbit anti-SARS-CoV-2 nucleocapsid antibody (Sino Biological, 40588-T62) at 1 : 1000 in PBS with 1 % BSA and goat anti-rabbit IgG Alexa Fluor 488 (Abcam, ab150077) at 1 : 1000 in PBS with 1 % BSA. Cell nuclei were counterstained with DAPI (Sigma, D9542). Images were acquired with the EVOS^TM^ FL imaging system AMEFC4300 (Invitrogen^TM^, WA, USA) using a 10× objective. The inbuilt software was used for image processing.

### Focus forming assay

The presence of the virus in the epithelial and endothelial channel washouts was quantified by FFA on Vero/E6 cells [ECACC 85020206], as previously described [21]. Differences between virus log-titres across times post-infection were compared with one-way ANOVA, using R 4.2 [22,24].

### Cytokine and chemokine analysis

Cytokine and chemokine secretion into the basolateral medium was quantified using multiplex Mesoscale Discovery System (MSD, Rockville, MD, USA, K15067M-1). IFN-β, IL-1α, IP-10, TNF-α, MIP-1α, IL-6, IL-8, GM-CSF, TRAIL and MCP-1 were quantified simultaneously using U-plex Human Biomarker Group 1 assay as per manufacturer’s instructions. The plates were read using the 1300 Meso QickPlex SQ 120 (MSD).

For statistical analysis, mixed effects models were used to estimate the effect of SARS-CoV-2 infection on cytokine concentrations over time. Time (as a discrete variable), treatment group and their interaction were included as fixed effects with random intercepts for each individual chip and for time point nested within chip. Models for each cytokine were estimated individually. Concentrations were transformed onto a log-scale prior to analysis. We used the lme4 (version 1.1-31, to estimate models) and emmeans (version 1.8.6, to obtain contrasts, confidence intervals and *P* values) for R version 4.2 [22,24]. Prior to analysis, data for several samples were removed if technical variation was unusually high. This was judged by visual inspection by comparing the differences between technical variates in all samples across all points in a qq-plot. A sensitivity analysis including all data points was conducted and shown not to affect the conclusions.

## Results

### Establishment of the alveolus-on-chip model

The alveolus MPS model was established by seeding primary human alveolar epithelial cells in the epithelial channel and human lung microvascular endothelial cells in the vascular channel of the chip. Once the epithelial monolayer was established, the ALI was set up in the epithelial channel. The steroid dexamethasone was omitted from the cell culture medium 1 day after establishing ALI. Stretch was introduced gradually over 2 days, from 2 to 5 % at 0.2 Hz, together with low airflow at 30 µl h^−1^. Using these conditions, ALI was maintained throughout the experiment accompanied with physiologically relevant stretch that mimics breathing (5 %, 0.2 Hz; Fig. S1). The chips were quality controlled by assessment of apparent permeability, which was found in the physiologically relevant range (papp < 5×10^−7^ cm s^−1^) characteristic of a tight epithelial barrier (see Fig. S2).

### Cytopathic effects of SARS-CoV-2 infection of alveolus-on-chips

Following infection with SARS-CoV-2, the MPS chips were observed daily with phase contrast microscopy. Morphological changes became widespread at 48 and 72 hpi, as shown by rounding of the infected cells, cell aggregation/clumping, syncytia formation and increased cytoplasmic vacuolization in the epithelial channel. Representative phase contrast images are shown in Fig. S3.

**Fig. 1.**
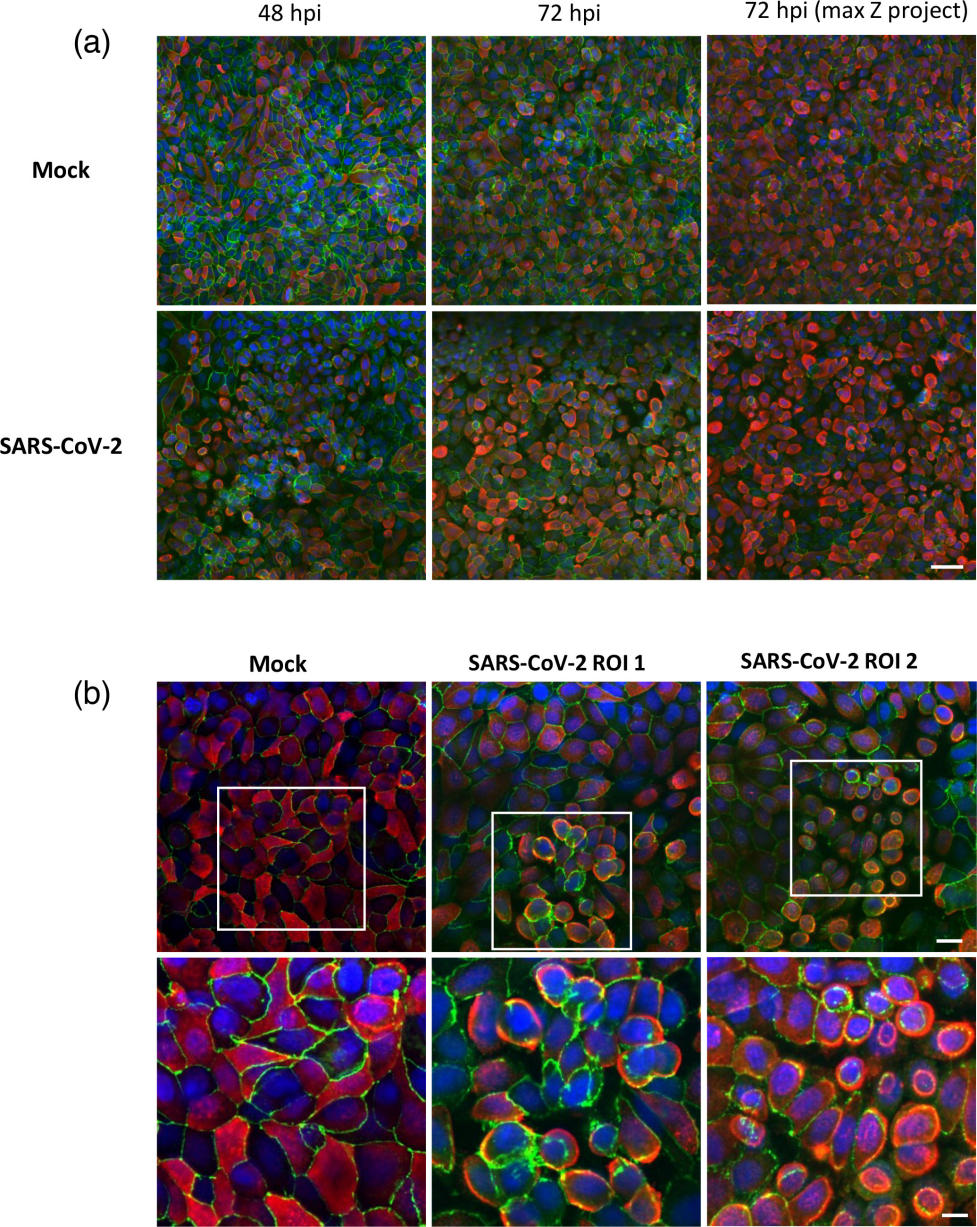
Immunofluorescence analysis of morphological changes triggered by SARS-CoV-2 infection of alveolus-on-chip. (**a**) Staining of AT2 (red) and OCLN (green) in the epithelium of alveolus-on-chips following mock-treatment or infection with SARS-CoV-2 at MOI of 1 at 48 and 72 hpi. Chips were counterstained with DAPI (blue). To aid visualization of AT2, the max projection function of the Z-stack is shown (panel a right). Scale bar 50 µm. (**b**) Focal view of the epithelium staining 72 hpi at higher magnification using 20× objective with 2× digital zoom (scale bar 20 µm, top panel). Regions of interest (ROI) using 4× digital zoom (scale bar 10 µm, bottom panel) showed the effects of SARS-CoV-2 on the shape of AT2 and OCLN distribution in more detail.

The effect of SARS-CoV-2 infection on the morphology of the epithelium at 48 and 72 hpi was then studied with immunofluorescence by staining alveolus-on-chips with antibodies specific for HT2-280, marker of AT2, and tight junction protein OCLN. While the control chips retained the typical pavement epithelial pattern (Fig. 1a, top panel), AT2 cells showed a more rounded appearance in the SARS-CoV-2-infected chips. This was observed at 48 hpi and became widespread at 72 hpi (Fig. 1a, bottom panel) and was mirrored by a change of the OCLN staining pattern at 48 and 72 hpi (Fig. 1a). A close-up examination of the rounded cells showed that OCLN pattern was either disrupted (Fig. 1b, ROI1) or redistributed in a way that mirrors the shape of rounded AT2 (Fig. 1b, ROI2). Together, these results indicate that SARS-CoV-2 infection of human alveolus MPS leads to the morphological and structural changes typically seen in human epithelial cells after naturally acquired SARS-CoV-2 [25].

### SARS-CoV-2 infection and propagation in the epithelium

**Fig. 2.**
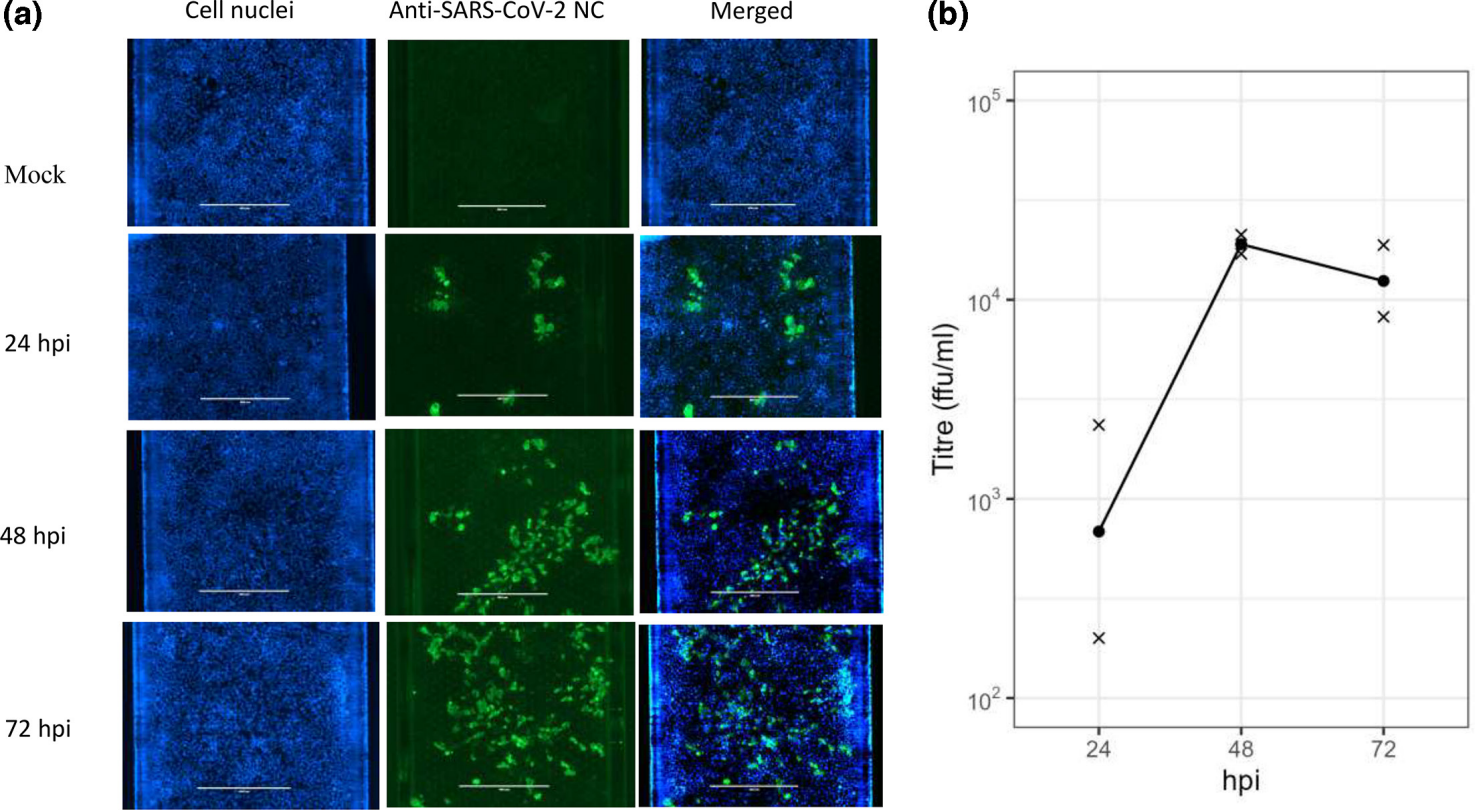
Detection of SARS-CoV-2 in epithelium and epithelial channel washout. (**a**) Staining of epithelial cells for SARS-CoV-2 nucleocapsid (NC, shown in green) at 24, 48 and 72 hpi. Cell nuclei were counterstained with DAPI (blue), scale bar 400 µm. (**b**) Titres of viable SARS-CoV-2 from epithelial washouts at 24 to 48 hpi and 72 hpi. Each value is an average of two SARS-CoV-2-treated chips per time point.

SARS-CoV-2 infection and replication in the alveolus-on-chips were first monitored by staining for nucleocapsid protein in the epithelium and titration of the released particles by FFA. The nucleocapsid protein was detected in the alveolar epithelial layer of all infected chips; at 24, 48 and 72 hpi. The presence of infected cells appeared to increase from 24 to 48 hpi and then remained unchanged until 72 hpi (Fig. 2a). No nucleocapsid protein was observed in the endothelial layer (data not shown).

The titration of viable virus in the washout of epithelial channel of alveolus-on-chips using FFA was consistent with these observations with a marked increase of SARS-CoV-2 from 24 to 48 hpi of approximately 30-fold before plateauing at 72 hpi (Fig. 2b; one-way ANOVA comparing log-titres across time points, *P* = 0.094). No virus was detected in the endothelial channel washout (Table S1). A positive control run on each plate, showed the expected titre. These results provide evidence of infection and active viral replication in the epithelium.

### Host responses to SARS-CoV-2 infection in the alveolus MPS model

**Fig. 3.**
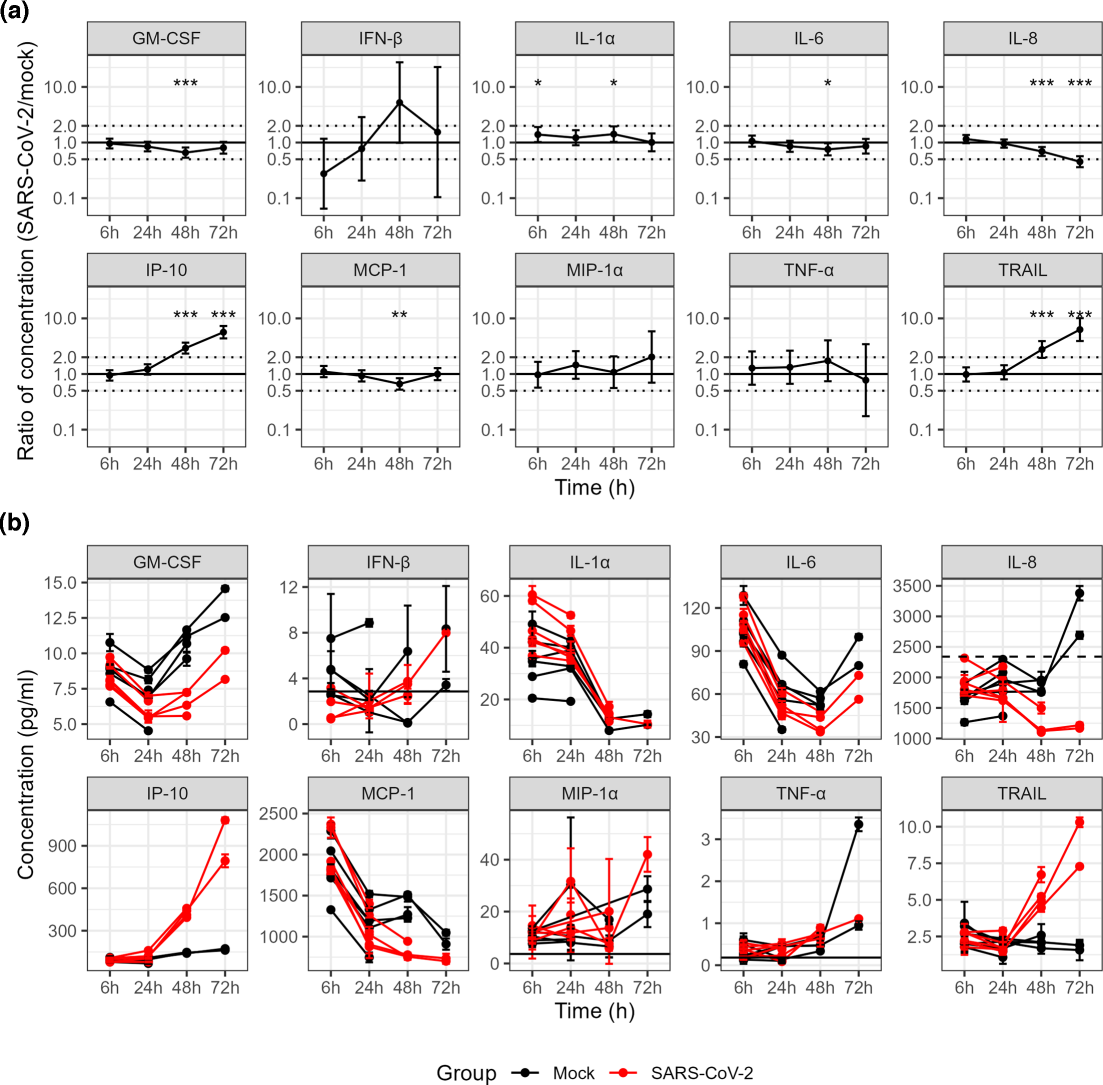
Cytokine, chemokine and ligand time-response to SARS-CoV-2 infection in alveolus-on-chips. (**a**) Estimated ratios for cytokine concentrations in endothelial effluents of SARS-CoV-2 versus mock-treated chips are shown at 6 hpi (*n*=6 chips per group), 24 hpi (*n*=6), 48 hpi (*n*=4) and 72 hpi (*n*=2). (**b**) Individual concentrations per chip for cytokine measurements are shown at the same time points. Error bars represent 95 % confidence intervals for estimates (panel a) and standard error of technical variation for individual data points (panel b). Horizontal lines represent upper (dashed) and lower (solid) detection limits when in the range of the observed data. Significance stars in panel a correspond to the null hypothesis that the log-ratio of concentrations between treatment groups is equal to zero, with *** corresponding to *P*<0.001, ** for *P*<0.01 and * for *P*<0.05.

To investigate the effect of SARS-CoV-2 infection on the host cells, we quantified the level of cytokines, chemokines and ligands (IFN-β, IL-1α, IP-10, TNF-α, MIP-1α, IL-6, IL-8, GM-CSF, TRAIL and MCP-1) using a multiplex immunoassay in alveolus-on-chips at 6, 24, 48 and 72 hpi and in mock-treated cells. Secretion of interferon gamma-induced Protein 10 (IP-10) and TNF-related apoptosis-inducing ligand (TRAIL) was shown to be significantly increased in SARS-CoV-2-infected chips in comparison with mock-treated chips (ratio = 5.7, *P* < 0.001 for IP-10 and 6.3, *P* < 0.001 for TRAIL at 72 hpi) (Fig. 3a). Conversely, concentrations of granulocyte-macrophage colony-stimulating factor (GM-CSF), monocyte chemoattractant protein-1 (MCP-1) and IL-6 were significantly lower in SARS-CoV-2-infected chips at 48 hpi than in mock-treated chips but not at 72 hpi (Fig. 3a). For IL-8, this trend was significant at both 48 and 72 hpi. There was no evidence for differences between groups at 6 or 24 hpi, except for IL-1α which was significantly increased at 6 and 48 hpi.

For MIP-1α, TNF-α and IFN-β, the concentrations measured were close to the lower limit of detection and characterized by high technical variation, leading to imprecise estimates of the effect of SARS-CoV-2 on these cytokines and chemokines (Fig. 3b).

## Discussion

This study reports the successful establishment of a human alveolus-on-chip microfluidic system to model SARS-CoV-2 infection of alveoli. The advantage of this system is to enable ALI which has been shown to be essential to preserve epithelial barrier properties [12] and stretching which mechanically mimics breathing and influences innate immune responses to viral infection [15]. Stretch was introduced progressively over 2 days together with apical airflow in order to maintain ALI upon initiation of physiologically relevant stretch.

Many patients with severe COVID-19 showed an uncontrolled overproduction of soluble inflammatory markers, and dexamethasone has been reported to suppress SARS-CoV-2-induced cytokine storm [26,27]. Since dexamethasone is used as a supplement for cell culture medium [16] to support alveolar cell differentiation and pulmonary function, this steroid was removed 6 days before inoculation of the chip with the virus to avoid unwanted suppression of the innate MPS immune response. This modification did not compromise the integrity of the cell barrier as shown by apparent permeability nor the successful SARS-CoV-2 infection.

Using these experimental conditions, infection with SARS-CoV-2 caused progressive cytopathic effects of epithelial cells, which became apparent 2 and 3 days post-inoculation. Rounded cell morphology of AT2 cell correlated with OCLN reorganization, and in some areas of the epithelium, OCLN was disrupted. OCLN has only recently been shown to be critical for SARS-CoV-2 internalization and cell-to-cell transmission [28]; however, cell rounding has been previously observed following SARS-CoV-2 infection in mammalian cell lines including Vero-E6, BGMK, MA-104, CV-1, RhMK, PK-15 and ST cells cultured under static conditions as reviewed recently [29] and lung organoids grown as monolayers [30], but to our knowledge, this has not been demonstrated using lung-on-chip model before.

Using this system, there was clear evidence of virus replication in the epithelial cells of all alveolar MPS chips inoculated with SARS-CoV-2 as shown by immunocytochemical staining for nucleocapsid protein, which is produced at high levels within infected cells [31]. More than a tenfold increase in detectable viable virus was also evident in the epithelial compartment from day 1 to day 2 post-infection and that high level remained stable until day 3. This is comparable with the timeline of infectious virus load shedding in patients which peaked around 1–2 days from the point of symptom onset [32]. SARS-CoV-2 replication was also observed *in vitro* where viral gene copy numbers increased from day 1 to day 2 post-inoculation and plateaued at day 3 in the epithelium of immortalized AT2 cells on alveolus-on-chip model [4], while a limited release was observed in the epithelial channel wash over 3 days post-infection in MPS chips containing primary alveolar and endothelial lung cells [3]. Differences in experimental designs including different SARS-CoV-2 variants, MOI, cell types, the presence/absence of stretch, and dexamethasone may have contributed to these discrepancies. Here, no viral replication was observed in the endothelial MPS channel, as also reported by the two alveolus-on-chip studies [3,4].

SARS-CoV-2 infection elicited an innate immune response in this human alveolus MPS with significant increase in IP-10, a chemoattractant for monocytes, TRAIL which induces apoptosis and to some extent IL-1α, a strong inflammatory cytokine associated with severe COVID-19 cases. IP-10 and TRAIL responses to SARS-CoV-2 mimic a natural infection and align with clinical data demonstrating a positive correlation between these cytokines and viral load [33]. In contrast, IL-8 and to some extent IL-6, which have been shown to be associated with disease severity of COVID-19 [34] and increased in other SARS-CoV-2 infection models [3,4], showed a reduction as a response to SARS-CoV-2 infection, which may be due to the absence of immune cells in our model. It is of note that although the chip design does permit the introduction of immune cells that can migrate across the membrane from one channel to the other as shown in Colon Intestine-Chip models of inflammation [35], the challenge in the context of infection would be to acquire primary cell types (epithelial, endothelial and immune cells) from the same donor or closely related HLA-matched donors to avoid immune responses due to HLA incompatibility. This is not a requirement for alveolar epithelial cells and lung endothelial cells as they are sequestered on different sides of the chip.

In conclusion, we have shown that, under the experimental conditions used in this work, the alveolar MPS chip SARS-CoV-2 infection model successfully recapitulates the early innate immune responses observed in the epithelium of naturally acquired SARS-CoV-2 infection in humans.

## supplementary material

10.1099/acmi.0.000814.v3 Uncited Supplementary Material 1.

## Abbreviations

ALI: air–liquid interface
AT1: alveolar type 1
AT2: alveolar type 2
BSL3: biosafety level 3
COVID-19: coronavirus 2019
FCS: foetal calf serum
FFA: focus forming assay
FITC: fluorescein isothiocyanate
GM-CSF: granulocyte-macrophage colony-stimulating factor
hpi: hours post-infection
IP-10: interferon gamma-induced protein 10
MOI: multiplicity of infection
MPS: microphysiological system
MSD: mesoscale discovery system
NC: nucleocapsid
OCLN: occludin
Papp: apparent permeability
SARS: severe acute respiratory syndrome
TRAIL: TNF-related apoptosis-inducing ligand

## References

[1] Giattino CR, Ritchie H, Ortiz-Ospina E, Hasell J, Rodés-Guirao L (2023). Excess mortality during the Coronavirus pandemic (COVID-19). https://ourworldindata.org/excess-mortality-covid.

[2] Gattinoni L, Coppola S, Cressoni M, Busana M, Rossi S (2020). COVID-19 does not lead to a “typical” acute respiratory distress syndrome. Am J Respir Crit Care Med.

[3] Thacker VV, Sharma K, Dhar N, Mancini G-F, Sordet-Dessimoz J (2021). Rapid endotheliitis and vascular damage characterize SARS-CoV-2 infection in a human lung-on-chip model. EMBO Rep.

[4] Zhang M, Wang P, Luo R, Wang Y, Li Z (2021). Biomimetic human disease model of SARS‐CoV‐2‐induced lung injury and immune responses on organ chip system. Adv Sci.

[5] Chen L-D, Zhang Z-Y, Wei X-J, Cai Y-Q, Yao W-Z (2020). Association between cytokine profiles and lung injury in COVID-19 pneumonia. Respir Res.

[6] Knudsen L, Ochs M (2018). The micromechanics of lung alveoli: structure and function of surfactant and tissue components. Histochem Cell Biol.

[7] Mason RJ (2020). Thoughts on the alveolar phase of COVID-19. Am J Physiol Lung Cell Mol Physiol.

[8] Jackson CB, Farzan M, Chen B, Choe H (2022). Mechanisms of SARS-CoV-2 entry into cells. Nat Rev Mol Cell Biol.

[9] Cantuti-Castelvetri L, Ojha R, Pedro LD, Djannatian M, Franz J (2020). Neuropilin-1 facilitates SARS-CoV-2 cell entry and infectivity. Science.

[10] Thunders M, Delahunt B (2020). Gene of the month: *TMPRSS2* (transmembrane serine protease 2). J Clin Pathol.

[11] Plebani R, Bai H, Si L, Li J, Zhang C (2022). 3D lung tissue models for studies on SARS-CoV-2 pathophysiology and therapeutics. Int J Mol Sci.

[12] Silva S, Bicker J, Falcão A, Fortuna A (2023). Air-liquid interface (ALI) impact on different respiratory cell cultures. Eur J Pharm Biopharm.

[13] Huh D, Matthews BD, Mammoto A, Montoya-Zavala M, Hsin HY (2010). Reconstituting organ-level lung functions on a chip. Science.

[14] Cao T, Shao C, Yu X, Xie R, Yang C (2022). Biomimetic alveolus-on-a-chip for SARS-CoV-2 infection recapitulation. Research.

[15] Bai H, Si L, Jiang A, Belgur C, Zhai Y (2022). Mechanical control of innate immune responses against viral infection revealed in a human lung alveolus chip. Nat Commun.

[16] Emulate Bio (2020). Alveolus lung-chip co-culture protocol. Document: EP180 v1.1. https://emulatebio.com/wp-content/uploads/2021/06/EP180_v1.1_Alveolus_Lung-Chip_Co-Culture_Protocol.pdf.

[17] Emulate Bio (2019). Protocol for emulate organ-chips: barrier function analysis. EP187 v1.0 – draft 10. https://emulatebio.wpenginepowered.com/wp-content/uploads/2021/06/EP187_v1.0_Barrier_Function_Analysis_Protocol.pdf.

[18] Caly L, Druce J, Roberts J, Bond K, Tran T (2020). Isolation and rapid sharing of the 2019 novel coronavirus (SARS-CoV-2) from the first patient diagnosed with COVID-19 in Australia. Med J Aust.

[19] Bewley KR, Coombes NS, Gagnon L, McInroy L, Baker N (2021). Quantification of SARS-CoV-2 neutralizing antibody by wild-type plaque reduction neutralization, microneutralization and pseudotyped virus neutralization assays. Nat Protoc.

[20] Schneider CA, Rasband WS, Eliceiri KW (2012). NIH Image to ImageJ: 25 years of image analysis. Nat Methods.

[21] Ryan KA, Bewley KR, Watson RJ, Burton C, Carnell O (2023). Syrian hamster convalescence from prototype SARS-CoV-2 confers measurable protection against the attenuated disease caused by the Omicron variant. PLoS Pathog.

[22] Bates D, Mächler M, Bolker B, Walker S (2015). Fitting linear mixed-effects models using lme4. J Stat Softw.

[23] Lenth RV, Buerkner B, Giné-Vázquez I, Herve M, Jung M (2023). emmeans: Estimated Marginal Means, aka Least-Squares Means. R package version 1.8.6. https://cran.r-project.org/web/packages/emmeans/index.html.

[24] R Core Team (2022). R: a language and environment for statistical computing. https://www.R-project.org.

[25] Stoyanov GS, Petkova L, Dzhenkov DL, Sapundzhiev NR, Todorov I (2020). Gross and histopathology of COVID-19 with first histology report of Olfactory bulb changes. Cureus.

[26] Morgan P, Arnold SJ, Hsiao N-W, Shu C-W (2021). A closer look at dexamethasone and the SARS-CoV-2-induced cytokine storm: in silico insights of the first life-saving COVID-19 drug. Antibiotics.

[27] Sharun K, Tiwari R, Dhama J, Dhama K (2020). Dexamethasone to combat cytokine storm in COVID-19: clinical trials and preliminary evidence. Int J Surg.

[28] Zhang J, Yang W, Roy S, Liu H, Roberts RM (2023). Tight junction protein occludin is an internalization factor for SARS-CoV-2 infection and mediates virus cell-to-cell transmission. Proc Natl Acad Sci USA.

[29] Pires De Souza GA, Le Bideau M, Boschi C, Wurtz N, Colson P (2022). Choosing a cellular model to study SARS-CoV-2. Front Cell Infect Microbiol.

[30] Tindle C, Fuller M, Fonseca A, Taheri S, Ibeawuchi S-R (2021). Adult stem cell-derived complete lung organoid models emulate lung disease in COVID-19. elife.

[31] Savastano A, Ibáñez de Opakua A, Rankovic M, Zweckstetter M (2020). Nucleocapsid protein of SARS-CoV-2 phase separates into RNA-rich polymerase-containing condensates. Nat Commun.

[32] Puhach O, Meyer B, Eckerle I (2023). SARS-CoV-2 viral load and shedding kinetics. Nat Rev Microbiol.

[33] Papan C, Argentiero A, Adams O, Porwoll M, Hakim U (2023). Association of viral load with TRAIL, IP-10, CRP biomarker signature and disease severity in children with respiratory tract infection or fever without source: a prospective, multicentre cohort study. J Med Virol.

[34] Chang Y, Bai M, You Q (2022). Associations between serum interleukins (IL-1*β*, IL-2, IL-4, IL-6, IL-8, and IL-10) and disease severity of COVID-19: a systematic review and meta-analysis. Biomed Res Int.

[35] Carman C, Kanellias M, Ramos D, Maniar K, Saud J (2022). Modeling inflammatory immune cell recruitment and response on human colon intestine-chip. J Immunol.

