# Supplementary material for: Modelling SARS-CoV-2 infection in a human alveolus microphysiological system
Source: Access Microbiol. 2024 Sep 11;6(9):000814.v3. doi: 10.1099/acmi.0.000814.v3 (PMC11652720; doi:10.1099/acmi.0.000814.v3)
Supplement: Uncited Supplementary Material 1. [file acmi-6-00814-s001.pdf]

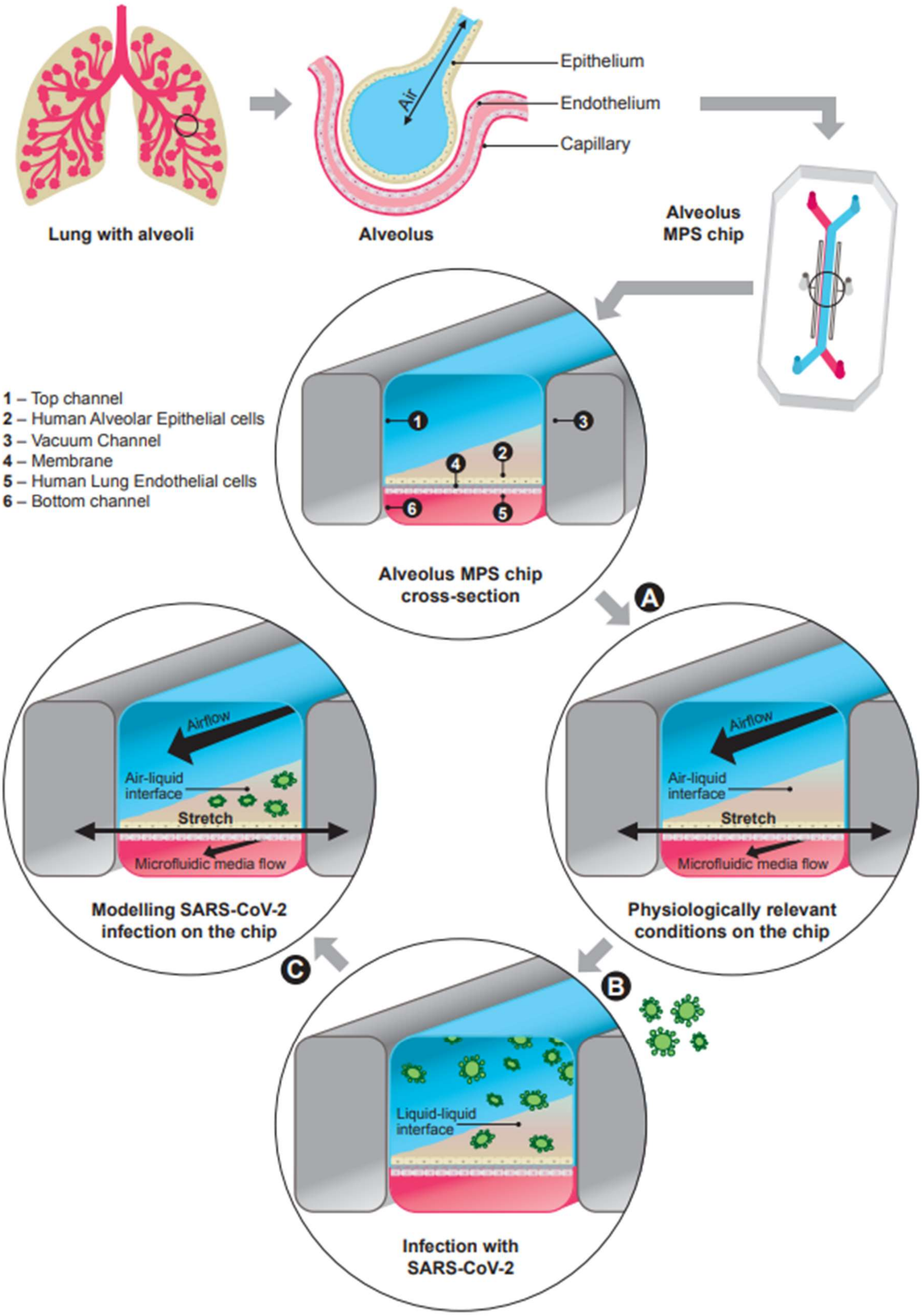

Fig. S1: **Physiologically relevant conditions on alveolus MPS chip model and infection with SARS-CoV-2.** (**A**) The schematics shows adaptation of the lung model that consists of human alveolar

epithelial and lung endothelial cells that are cultured under physiologically relevant conditions including: (i) airflow in the top channels which allows the alveolar epithelial cells to be maintained in air-liquid interface, (ii) stretch that mimic breathing and (iii) microfluidic flow of medium in bottom channel that feeds the cells. (**B**) The virus was introduced to the top channel of the chip with alveolar epithelial cells and incubated under static conditions. Afterwards, the air-liquid interface was re-established and the airflow, stretch and airflow reintroduced (**C**).

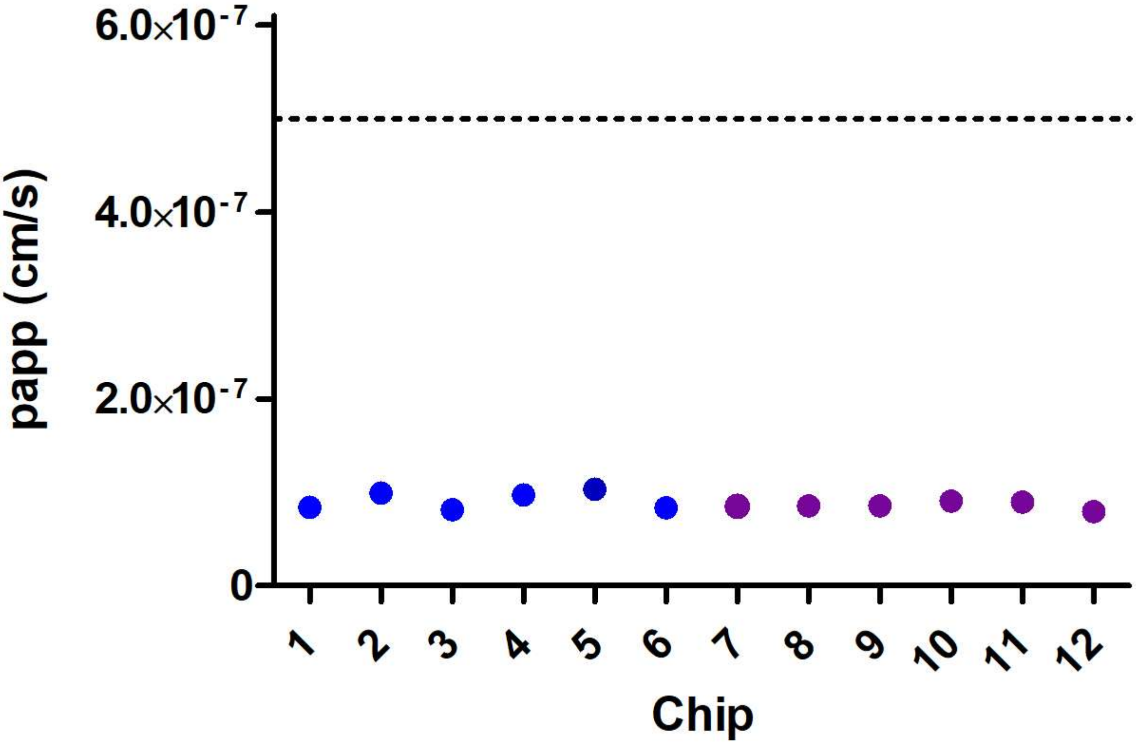

Fig. S2: **Apparent permeability (papp) of alveolus-on-chips on day 4 of ALI.** Dotted line at 5 x 10^{-7} cm/s is the upper cut-off value for quality control check of physiologically relevant permeability. Chips 1-6 (blue) were mock-treated and chips 7-12 (violet) were treated with SARS-CoV-2 on d7 of ALI.

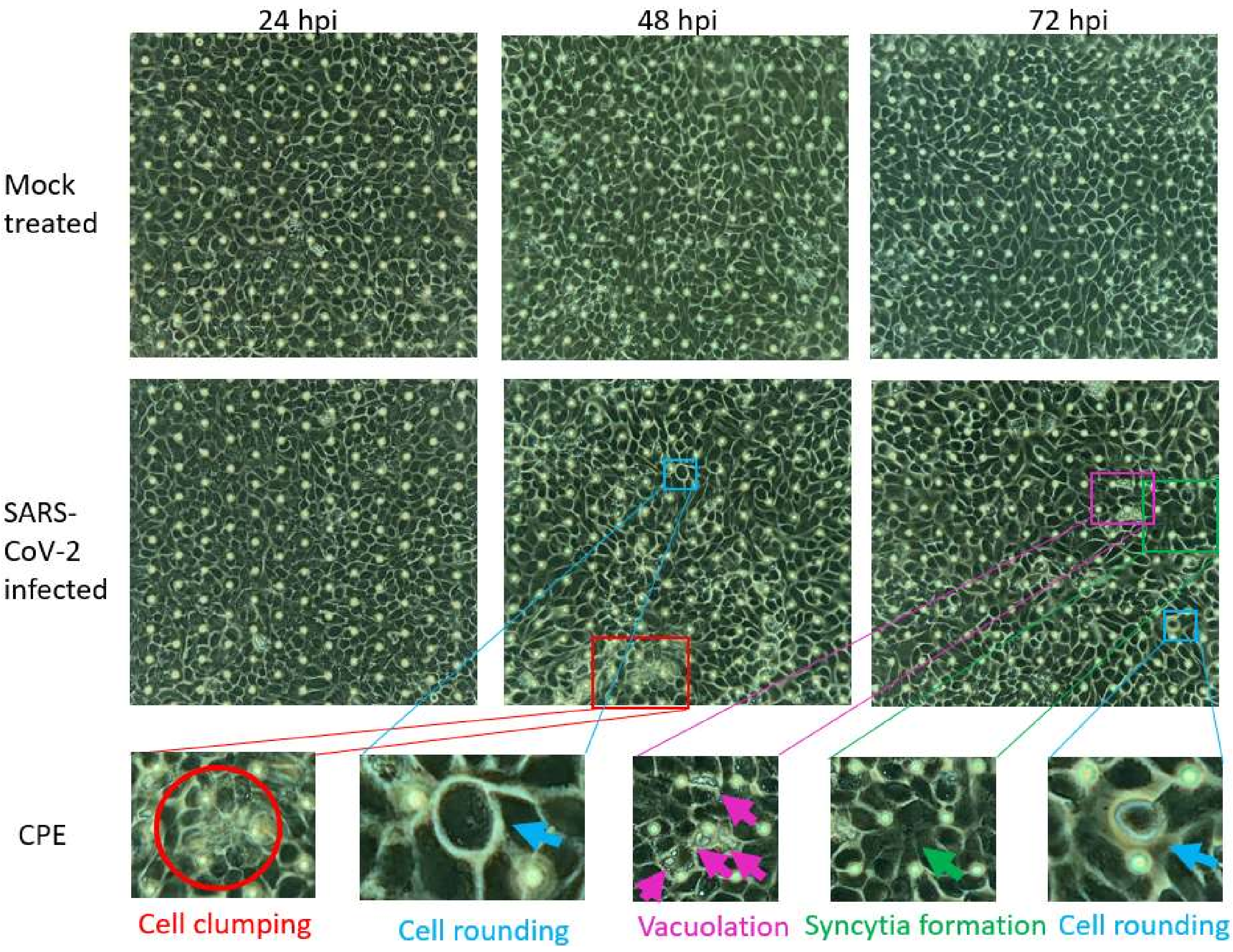

Fig. S3: Phase contrast imaging of alveolar epithelial cells infected with SARS-CoV-2. Images were acquired 24, 48 and 72 hpi with 20x objective. Top panel shows mock treated chips, middle panel SARS-CoV-2 infected chips and bottom panel cytopathic effects (CPE) observed. These include cell clumping, cell rounding, vacuolation and syncytia formation.

Table S1 – Results from focus forming assays measuring viable virus from apical and basal channels washouts of chips infected with SARS-CoV-2 (chips 7-12) sampled at 24, 48 and 72 hpi. A positive control was run on each plate (expected titre $3x10^6$ ffu/ml).

Sampling	Chip/control	Time point of sampling	Titre (ffu/ml)
Apical washout/ epithelial channel (plate 1)	7	24 hpi	2.4×10^3
	8	24 hpi	2.0×10^2
	9	48 hpi	2.1×10^4
	10	48 hpi	1.7×10^4
	11	72 hpi	8.2×10^3
	12	72 hpi	1.9×10^4
	Positive control	N/A	2.0×10^6
Basolateral washout/ endothelial channel (plate 2)	7	24 hpi	ND
	8	24 hpi	ND
	9	48 hpi	ND
	10	48 hpi	ND
	11	72 hpi	ND
	12	72 hpi	ND
	Positive control	N/A	$2.1x10^6$

*ND = not detected
